# Evolution of the Immune Response against Recombinant Proteins (TcpA, TcpB, and FlaA) as a Candidate Subunit Cholera Vaccine

**DOI:** 10.1155/2017/2412747

**Published:** 2017-01-16

**Authors:** Neda Molaee, Ghasem Mosayebi, Alireza Amozande-Nobaveh, Mohammad Reza Soleyman, Hamid Abtahi

**Affiliations:** ^1^Department of Microbiology and Immunology, School of Medicine, Arak University of Medical Sciences, Arak, Iran; ^2^Molecular and Medicine Research Center, Department of Microbiology and Immunology, School of Medicine, Arak University of Medical Sciences, Arak, Iran

## Abstract

*Vibrio cholerae* is the causative agent of cholera and annually leads to death of thousands of people around the globe. Two factors in the pathogenesis of this bacterium are its pili and flagella. The main subunits of pili TcpA, TcpB, and FlaA are the constituent subunit of flagella. In this study, we studied the ability of pili and flagella subunits to stimulate immune responses in mice. After amplification of TcpA, TcpB, and FlaA genes using PCR, they were cloned in expression plasmids. After production of the above-mentioned proteins by using IPTG, the proteins were purified and then approved using immunoblot method. After injection of the purified proteins to a mice model, immune response stimulation was evaluated by measuring the levels of IgG1 and IgG2a antibody titers, IL5 and IFN-*γ*. Immune response stimulation against pili and flagella antigens was adequate. Given the high levels of IL5 titer and IgG1 antibody, the stimulated immune response was toward Th1. Humoral immune response stimulation is of key importance in prevention of cholera. Our immunological analysis shows the appropriate immune response in mice model after vaccination with recombinant proteins. The high level of IL5 and low level of IFN-*γ* show the activation of Th2 cell response.

## 1. Introduction

*Vibrio cholerae* is a noninvasive, Gram-negative bacterium that causes acute gastroenteritis in humans. Millions of people around the globe are suffering from cholera, and annually more than 100,000 cases of mortality are reported from this disease [1, 2].

The main structures of the bacteria which play an important role in the disease process include bacterial toxin, cell wall, flagella, and pili [3, 4]. Pilus plays a major role in the development and progression of infection. Several proteins are involved in formation of pilus, but only a limited number of them are on the bacterial cell surface. The most important of these proteins are toxin coregulated pilus subunit A (TcpA) and TcpB [5]. Several studies have demonstrated that antibodies against TcpA and TcpB can affect immunogenicity by reducing* Vibrio cholerae* colonization. A study performed by Rollenhagen et al. (2006) showed that immunization of mice with pilin proteins can induce protective immunity [6, 7].

TcpA is able to stimulate Th1-type immune responses, so that Tcp antigens have the ability to stimulate IL-4 cytokines and produce IgG1 antibodies. There is limited information on TcpB; this subunit is assumed to be effective in immune responses to cholera. Also, flagella-A (Fla-A) of* Vibrio cholerae* has a main role in pathogenicity of disease [8, 9].

Although bacterial pili and flagellar antigens are of major significance in the development and progression of cholera, there is a scarcity of studies on the type of immune responses induced by these antigens. In addition, when simultaneously inoculated, the effect of each of these antigens on the other has not been investigated yet. Therefore, the aim of this study is evaluation of immune responses against recombinant proteins TcpA, TcpB, and FlaA or combination of them in animal model.

## 2. Materials and Methods

### 2.1. Bacteria and Sera

*V. cholerae serotype Inaba* (a gift from the Pasteur Institute of Iran) was grown on TCBS (thiosulfate-citrate-bile salts-sucrose agar, Merck, Germany) for 24 hours. For recombinant protein production, prokaryotic expression vector pET32a (Novagene) and pGEX4T1 were used.* E. coli* strain DH5*α* (Stratagene) was used for initial cloning and* E. coli* BL21 (DE3) pLysS and* E. coli* BL21 were used as host strains for protein production.

The required antibiotics (ampicillin and chloramphenicol) were added to LB media according to the reference recommendation [10]. We received standard rabbit anti-*V. cholerae* sera from Tarbiat Modares University (Department of Microbiology, Tehran, Iran). All chemicals were obtained from Merck Co. (Germany).

### 2.2. Gene Amplification, Expression, and Purification of Recombinant Proteins

*V. cholerae* chromosomal DNA was prepared according to standard CTAB/NACL method [11].

Primers were designed according to published sequence for TcpB (accession number: FJ209011), TcpA (accession number: U09807), and FlaA (accession number: Af019213) of* V. cholerae* as follows: TcpB: forward: 5′TCGGATCCATGAGAAAATACCAA3′ and reverse: 5′ACTCGAGATTTTCACACCATTGA3′; TcpA: forward: 5′-AGGGATCCATGACATTACTCGAAG-3′ and reverse: 5′-AACTCGAGGCTGTTACCAAATGC-3′; FlaA: forward: 5′CTGGATCCATGACCATTAACGTAAA3′ and reverse: 5′CCTCGAGCTGCAATAACGHAGATT3′. All primers contained BamHI (forwards) and XhoI (reverses) sites, respectively. The restriction enzyme sites (underlined) were added to the primers for subsequent cloning procedure. PCR amplification was performed (separately) and was analyzed by horizontal agarose gel electrophoresis in 1x TBE buffer and visualized by ethidium bromide staining on UV transilluminator.

The PCR products were digested with BamHI and XhoI and ligated to pGEX4T1 (TcpA-pGEX4T1) and pET32a (TcpB-pET32a and FlaA-pET32a), which were digested by the same restriction enzymes, using T4 DNA ligase at 16°C overnight.* E. coli* BL21 and* E. coli* BL21 (DE3) pLysS competent cells were prepared by calcium chloride method and were used for transformation of TcpA-pGEX4T1, TcpB-pET32a, and FlaA-pET32a plasmids, respectively [12].

*E. coli* BL21 (DE3) pLysS (transformed by TcpB-pET32a and FlaA-pET32a plasmids) and* E. coli* BL21 (transformed by TcpA-pGEX4T1) were grown in 2 mL nutrient broth medium being supplemented with ampicillin (100 mg/mL) and chloramphenicol (35 mg/mL for* E. coli* BL21 (DE3) pLysS) on shaking incubator overnight at 37°C. In the next day, 500 *μ*L of culture was inoculated in 50 mL of nutrient broth medium 0.5 g yeast extract, 1 g bactopepton, 0.1 g glucose, 0.5 g NaCl, 0.05 g KCl, 0.025 g MgCl_2_·6H_2_O, 0.025 g CaCl_2_, 0.25 g nutrient broth, ampicillin (100 mg/mL), and chloramphenicol (35 mg/mL for* E. coli* BL21 (DE3) pLysS) at 37°C with vigorous agitation at 220 rpm. The cells grew until the OD (optical density) at 600 nm reached 0.6. Expression of the recombinant proteins was induced by the addition of isopropyl-*β*-D-thiogalactopyranoside (IPTG, Fermentas) to a final concentration of 1 mM and incubated for four hours [13].

TcpB and FlaA were purified using Ni-NTA column (Qiagen) and TcpA was purified by GST column (Bio-Sciences) according to manufacturer's instructions. The purified protein was dialyzed twice against PBS (pH 7.5) at 4°C overnight. The quality and quantity of purified recombinant proteins were analyzed by SDS-PAGE (15%) and Bradford methods, respectively.

### 2.3. Immunoblot Analysis

The integrity of the purified recombinant proteins was confirmed by Western blot analysis. Western blotting was performed according to the standard protocol [14] using sera from rabbit immunized against* Vibrio cholerae* and negative sera (as the negative controls) as the primary antibody at 1 : 100 dilution and HRP-conjugated (horseradish peroxidase) goat anti-rabbit IgG (Abcam, United Kingdom) at 1 : 2500 dilution in 1x TBST buffer (10x: 15 mMNaCl, 10 mMTris-HCl (pH = 7.4), 0.1% Tween 20) as secondary antibody. The reactions were developed by diaminobenzidine (DAB) solution (Roche, Germany).

### 2.4. Evaluation of the Immune Responses against Recombinant Proteins

To investigate immune responses against the recombinant proteins, six groups of BALB/c male mice were studied (*n* = 10) with a mean age of six weeks and weight of about 60 g. The groups were injected with poly(butylene succinate) (PBS, negative control), TcpA, TcpB, TcpA+TcpB, FlaA, and TcpA+TcpB+FlaA. Injections were performed intradermally in three doses with an interval of two weeks. The first dose of protein injection was administered with complete Freund's adjuvant, and, in the next sessions, the proteins were injected with incomplete Freund's adjuvant. Each mouse was injected with 75 *μ*g of the proteins per injection.

### 2.5. Separation of Mononuclear Cells from Spleen

At day 28 after immunization, mice were sacrificed by CO_2_ inhalation, and the spleens were removed. Spleens were washed in RPMI (modification with 5 mM HEPES, 100 U/mL of penicillin, and 100 *μ*g/mL of streptomycin (all from Gibco, Life Technologies, Inc., Gaithersburg, MD). The spleens were punctured repeatedly with a pair of forceps to release the spleen cells. Low-density mononuclear cells were collected after standard separation on Ficoll-Paque (Pharmacia Biotech, Uppsala, Sweden) and washed in RPMI with 10% heat-inactivated fetal bovine serum (FBS). The number of viable cells was assessed by trypan blue exclusion. The cells were then resuspended in RPMI supplemented with 10% FBS and used for proliferation assay and cytokines detection.

### 2.6. Cytokines Assay

The levels of cytokines (IL5 and IFN-*γ*) were measured in serum and supernatant of MNCs (mononuclear cells) cultured. MNCs isolated from spleen of mice at day 28 after immunization were incubated in 1 mL cultures at a density of ~2 × 106 cells/mL in the presence or absence of specific recombinant protein (20 *μ*g/mL) and placed in a 5% CO_2_, 37°C incubator for 72 h. For quantitative IFN-gamma and IL5 productions, supernatants were collected and the amounts of IFN-gamma and IL5 in the supernatants were quantified by ELISA kit (R&D Systems) according to the manufacturer's protocol. 96-well plates were coated with anti-mouse IFN-gamma and IL5 antibody in coating buffer and incubated overnight. After blocking, samples and standards at 1 : 2 serial dilutions of IFN-gamma and IL10 (standard curve) were added to the plates and incubated for 60 min at room temperature. After that, biotinylated anti-mouse IFN-gamma and IL5 mAbs were added and incubated for another 60 min. HRP-conjugated streptavidin was then added for 30 min. After further washings, TMB substrate was added to incubate for 30 min, followed by addition of 0.18 M H_2_SO_4_ solution to stop the reaction and reading at 450 nm was obtained.

### 2.7. Measurement of IgG1 and IgG2a Antibodies

ELISA method was employed to measure IgG1 and IgG2a antibodies in the vaccinated animals. In this method, 10 *μ*g of recombinant proteins TcpA, TcpB, and FlaA was first bound in 96-well plate for 4 h. After washing, the proteins were kept at 4°C. After incubation with 2% BSA solution for an hour, blocking stage was performed. Thereafter, 100 *μ*L of serum from the vaccinated mice was added to each 96-well microplate (NUNC). The plates were kept at 37°C for 2 h. After washing, 50 *μ*L of 1/10000 diluted antibodies against IgG1 and IgG2a conjugated classes was added to HRP (Abcam, UK) and they were kept at 37°C for 2 h. Finally, light absorption at wavelength of 490 nm was determined by adding 100 *μ*L of o-phenylenediamine (OPD) substrate solution to each well and incubation for 10 min.

### 2.8. Proliferative Response Check by MTT

Proliferation was checked by MTT method [15]. A total of 1-2 × 10^3^ cells/well in 100 *μ*L RPMI 1640 supplemented with 10% FBS were stimulated with 20 *μ*g/mL specific antigen (TcpA, TcpB, and FlaA) or 1 *μ*g/mL PHA (phytohemagglutinin) concentration (as a mitogen) used for T-cell activation and proliferation. The plates were then placed in a 5% CO_2_, 37 °C incubator for 72 h. Ten microliters of 5 mg/mL MTT (3,4,5-dimethylthiazol-2-yl)-2,5-diphenyltetrazolium bromide, Sigma, Germany) was added to the cells, followed by incubation for 4 h. After centrifugation, the medium was removed, and 200 *μ*L of DMSO was added to each well. Then, the optical density at 570 nm was measured by microtiter plate reader (Stat Fax 2100, USA). The experiments were performed in triplicate sets. Blastogenic responses for the MTT assay were expressed as a mean stimulation index (SI) by dividing OD values of stimulated cells (C) minus relative cell numbers of unstimulated cells (C) by relative OD values of unstimulated cells. SI = (C − C)/C_MTT_.

### 2.9. Statistical Analysis

Statistical analysis included independent sample *t*-test to evaluate differences between variables in the groups. Comparisons between groups were assessed using Mann–Whitney* U* test. Data were analyzed by SPSS software version 16.0 (SPSS, Inc., Chicago, Illinois, USA) and were represented as mean ± SD. *P* values < 0.05 were considered statistically significant.

## 3. Results

### 3.1. Quality of Expressed Protein

**Figure 1:**
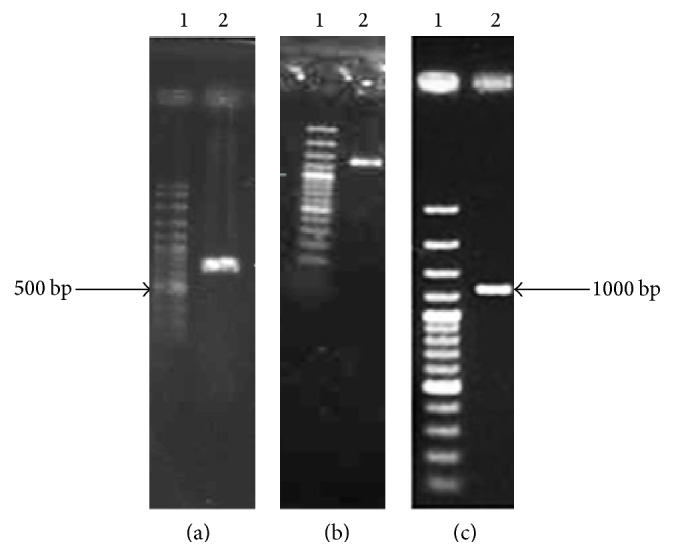
Results of amplification of TcpA, TcpB, and FlaA genes. (a): lane 1: DNA marker 100 bp (Fermentas); lane 2: TcpA gene PCR result (598 bp). (b): lane 1: DNA marker 100 bp; lane 2: TcpB gene PCR result (1295 bp). (c): lane 1: DNA marker 100 bp; lane 2: FlaA gene PCR result (1139 bp).

The results of polymerase chain reaction (PCR) consisted of gene fragments in the desired size (Figures 1(a), 1(b), and 1(c)). Sequencing and analysis using BLAST indicated the accuracy of the obtained sequence. The amplified DNA fragments (TcpA, TcpB, and FlaA) were cloned in pET32a and pGEX4T1 vectors.

TcpA, TcpB, and FlaA proteins were produced in* Escherichia coli*. The produced FlaA and TcpA proteins were purified using Ni-NTA kit and TcpB protein was purified using GST-Sepharose kit; the produced proteins were in the expected size. The amounts of proteins produced after purification and dialysis (against PBS buffer, in pH 7.5) were measured to be 2.1, 2, and 1.5 mg/mL. Western blotting of the produced proteins is exhibited.

**Figure 2:**
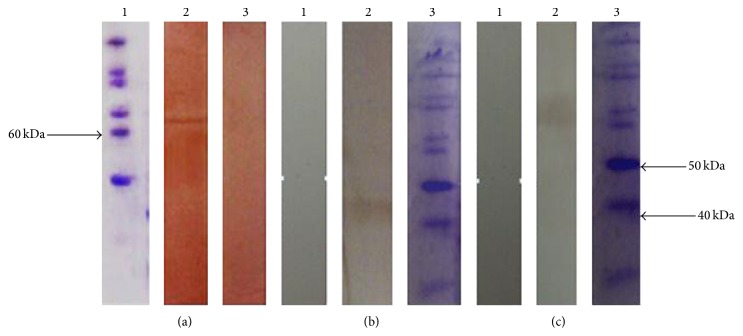
Western blot analysis of the recombinant proteins (a, b, c). (a) lane 1: protein marker (Fermentas); lane 2: interaction between serum of immunized rabbit and purified recombinant FlaA protein. (b) lane 1: protein marker; lane 2: interaction between serum of immunized rabbit and purified recombinant TcpA protein. (c) lane 1: protein marker; lane 2: interaction between serum of immunized rabbit and purified recombinant TcpB protein.

According to the result of the Western blotting, the purified proteins reacted against the serum of rabbit infected with* Vibrio cholerae*. Nevertheless, no reactions were observed in the serum of the healthy controls and the normal rabbit serum in the Western blotting (Figures 2(a), 2(b), and 2(c).

### 3.2. Cytokine Response (Serum and Supernatant)

**Figure 3:**
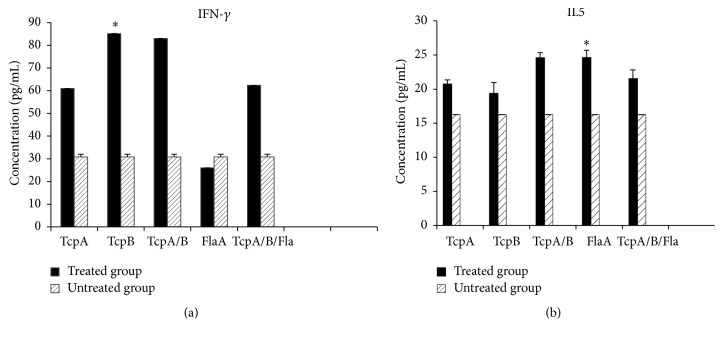
Quantitative ELISA analysis of IFN-*γ* (a) and IL5 (b) measured in the supernatant of the MNCs isolated from the spleen of mice immunized with specific recombinant proteins. Significant differences were designated as ^*∗*^*P* < 0.05.

**Figure 4:**
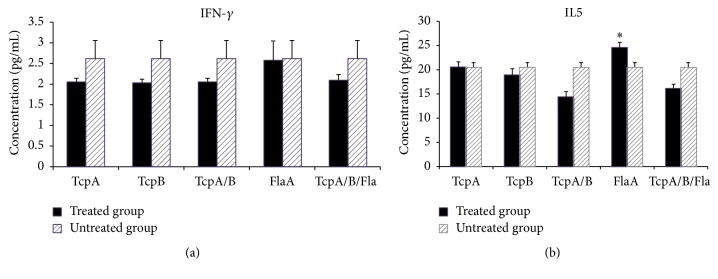
Quantitative ELISA analysis of IFN-*γ* (a) and IL5 (b) measured in the serum of mice immunized with specific recombinant proteins. Significant differences were designated as ^*∗*^*P* < 0.05.

The type of cytokines produced in the lymphocyte culture (serum and supernatant) of the mice immunized by recombinant proteins was determined three weeks after the last inoculation. Figures 3(a) and 3(b) show the levels of IFN-gamma and IL5 produced in the cultured lymphocytes (supernatant) of the immunized animals. As noted in Figure 3(a), IFN-gamma level increased in all of the studied groups except the FlaA. In Figure 3(b) curve, IL5 level is higher in all the experimental groups compared to the untreated group. This increase, particularly in FlaA and TcpA/B groups, is higher than the other groups. In serum (Figures 4(a) and 4(b)), IFN-gamma level in the serum was not significant. In Figure 4(b) curve, IL5 level is higher in FlaA compared to the untreated group.

### 3.3. Antibody Response

**Figure 5:**
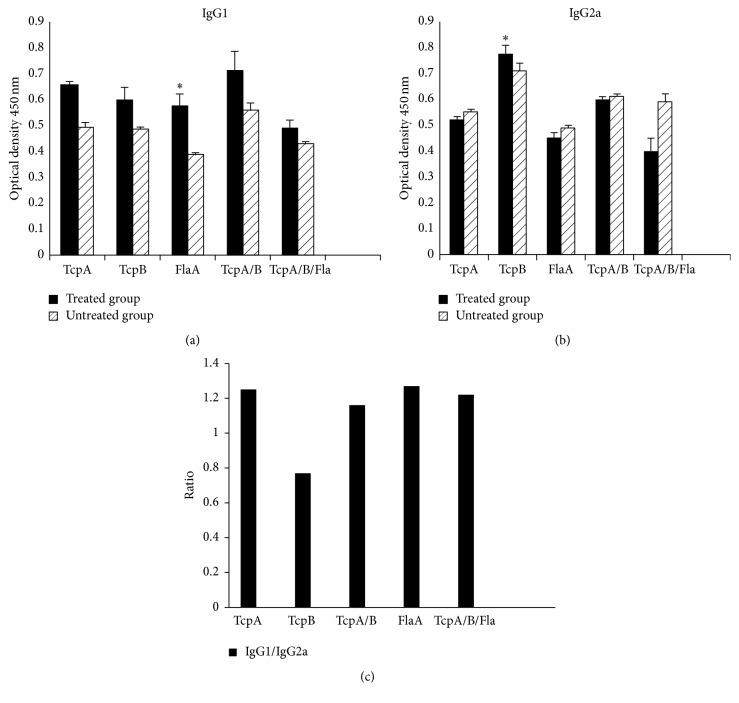
Measuring IgG1 (a) and IgG2a (b) and IgG1/IgG2a (c) antibodies in the vaccinated mice using ELISA method three weeks after immunization. Significant differences were designated as ^*∗*^*P* < 0.05.

Serum collected after the last vaccination was evaluated against the purified recombinant proteins TcpB, TcpA, and FlaA with regard to IgG2a and IgG1 antibody titers using ELISA method. As seen in Figures 5(a) and 5(b), the level of IgG1 antibody titer in all the vaccinated groups, compared to the untreated group, is higher than IgG2a antibody titer. In TcpB IgG2a antibody titer level is higher than the untreated group. However, IgG2a antibody titer level in groups receiving TcpA/B+FlaA is lower than the untreated group. IgG1 to IgG2a ratio is shown in Figure 5(c).

### 3.4. Lymphocyte Proliferation

**Figure 6:**
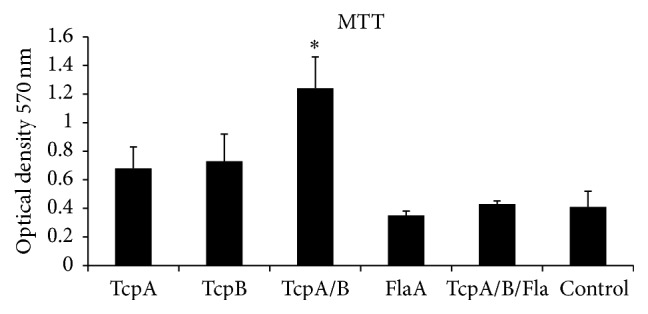
The MTT assay of the MNCs of BALB/c male immunized with specific recombinant proteins. Significant differences were designated as ^*∗*^*P* < 0.05.

According to the outcomes, the highest cell response in the experimental groups is observed in the TcpA+TcpB group. The lowest rate of cell proliferation is noted in the FlaA group. Results of MTT can be observed in Figure 6.

## 4. Discussion

The results show that pili and flagella antigens of* Vibrio cholerae* can cause strong humoral immune responses after immunization. However, TcpB antigen drives immune response away from the humoral immune response.

Cholera is an acute diarrheal infection caused by* Vibrio cholerae*. Following ingestion of contaminated water or food and after colonization of the bacteria in the small intestine, toxins are secreted by the bacteria, and, therefore, the patient loses great amounts of water and electrolytes. Given the high rates of mortality of the disease, prevention is crucial, especially in high-risk areas [16].

A large number of live attenuated or inactivated vaccines have already been studied, but none of these types of vaccines have been able to successfully prevent this disease, especially in endemic areas. Accordingly, more attention is drawn to subunit vaccines of* Vibrio cholerae* [17]. Adhesion and motility play a very important role in the process of* Vibrio cholerae* infectivity [18].

Pili and flagella are two important structures of* Vibrio cholerae* which help with adhesion and motility of the bacterium. TcpA and TcpB are the main subunits of* pilus* in* Vibrio cholerae* which can be seen on its outer surface [19].

TcpA is the main subunit of* pilus* in* Vibrio cholerae*. This antigen plays the main role in attachment of the bacterium to intestinal cells. In studies on synthetic version of the protein, it has been shown that peptides made from TcpA induce protective antibodies in mice and that these antibodies have a high protective effect. However, the level of antibody titer against this protein is not high in those vaccinated with live attenuated or inactivated vaccines, which is due to the sharp decline of this antigen in* Vibrio cholerae* culture [20].

TcpB is another component of* pilus* which helps with attachment of* Vibrio cholerae* to intestinal cells. Despite studies on the immunogenicity of TcpA, there are only few studies on the immunogenicity of the subsidiary subunit of TcpB. Only the study by Kiaie et al. showed that TcpB antibody exists in cholera patients as well as in animals vaccinated with killed bacteria [21].

In addition to adhesion, motility of* Vibrio cholerae* is considered as one of the most important indicators of bacterial pathogenicity. Flagella play a key role in the bacterium's access to the bacterial colonization location. As it was proven, mutant is not capable of causing infection without flagella. According to former studies, flagella are able to enhance expression of some pathogenicity genes such as the gene encoding the cholera toxin (CT), genes encoding pilus (TCP), and other genes involved in this disease. Therefore, flagella in* Vibrio cholerae* are considered as a suitable subject for immunological studies [22–24].

FlaA is the most important constituent of the outer surface of flagella. This protein is recognized by the normal immune response system, such that the body's first line of defense would respond against the bacterium. In a study conducted in 2008, it was revealed that recombinant flagella of* Vibrio cholerae* are able to stimulate IL8 production through the activation of Toll-like receptor 5 (TLR5) and nuclear factor kappa B. Thus, flagella of* Vibrio cholerae* are considered one of the main targets of the immune system and act as a ligand for Toll-like receptors, that is, TLR5, of the host immune cells. TLR5 stimulation by various pathogens leads to the activation of the innate immune response and, in turn, adaptive immunity [25].

In immunogenicity studies on pili and flagella antigens, the effects of immunogenicity of antigens have been studied separately. In the present study, the immunogenicity of these antigens was studied. The vaccinated groups (six groups) included mice vaccinated with TcpA, TcpB, FlaA, TcpA+TcpB, and TcpA+TcpB+FlaA. After the full course of immunization, humoral immunity and cellular immunity were evaluated in the groups.

Evaluation of cytokine responses after stimulation of spleen cells with recombinant proteins TcpA, TcpB, and FlaA exhibited the ability of these proteins to produce IFN-gamma and IL5. Stimulation level of the production of IFN-gamma after stimulation of lymphocytes of mice immunized by the proteins TcpA and TcpB can be observed. Furthermore, the lowest level of IFN-gamma production is observed in mice immunized with FlaA. However, in mice immunized with all the three proteins (TcpA+TcpB+FlaA), the level of IFN-gamma slightly increased compared to the FlaA group.

Measurement of serum IFN-gamma in the immunized mice showed that there was no significant different between the groups.

IL5 production rate in the vaccinated groups showed that the highest rate of cytokine production was observed in mice immunized with FlaA protein. The level of IL5 in serum and cell culture in the vaccinated animals increased. Statistical results also demonstrated that *P* value obtained from the FlaA group was less than 0.05. IL5 in cell culture and serum of mice given FlaA was more than the level of this cytokine in the untreated group. Nevertheless, the level of IL5 in the cell culture of the TcpB group was lower than the other groups. In addition, the level of IL5 in the serum of TcpB and TcpA+TcpB and TcpA+TcpB+FlaA groups was less than the untreated group.

Thus, according to the obtained results regarding cytokine (in the lymphocyte culture), it is indicated that the immune system in TcpA and FlaA groups tends toward humoral immune responses, while TcpB drives the immune responses away from humoral immunity.

The levels of IgG1 and IgG2a antibody titers in the studied groups partly confirm the cytokine results. IgG1 antibody titer increased in all the studied groups. Moreover, IgG1 titer was high in the groups receiving FlaA and TcpA proteins, such that the IgG1 to IgG2a ratio was more than one in these groups. Increased level of IgG1 titer was also noted in the TcpB group. The promoted level of antibody titer can be observed even in the groups that received several antigens with TcpB.

High levels of IgG2a titer can be clearly observed in the TcpB group. Additionally, this increase can be observed in the groups where the antigen was injected with other antigens, especially in the TcpA+TcpB group. In the FlaA+TcpA/B group, the level of this antibody was less than the untreated groups.

IgG1 to IgG2a ratio in the studied groups demonstrates that, in the groups inoculated with the FlaA and TcpA antigens, this ratio was higher than one. However, this ratio was less than one in the TcpB group. Combination of TcpB with other antigens lowered this ratio, compared to the other groups.

High levels of IgG2a titer and IFN-gamma are observed in the groups vaccinated with TcpB. Therefore, it is inferred that TcpB mostly plays a role in stimulating cellular immune responses.

According to the results, it can be concluded that FlaA and TcpA stimulate immune responses toward Th2; however, high levels of TcpB mostly direct these responses toward Th1. Since using vaccines consisting of pathogen subunits is more efficient when two or more subunits are used, the use of FlaA and TcpA antigens in* Vibrio cholerae* vaccination can cause higher immunogenicity.
